# PTV margin determination in conformal SRT of intracranial lesions

**DOI:** 10.1120/jacmp.v3i3.2561

**Published:** 2002-06-01

**Authors:** Brent C. Parker, Almon S. Shiu, Moshe H. Maor, Frederick F. Lang, H. Helen Liu, R. Allen White, John A. Antolak

**Affiliations:** ^1^ Department of Radiation Physics The University of Texas M. D. Anderson Cancer Center 1515 Holcombe Boulevard Houston Texas 77030; ^2^ Department of Radiation Oncology The University of Texas M. D. Anderson Cancer Center 1515 Holcombe Boulevard Houston Texas 77030; ^3^ Department of Neurosurgery The University of Texas M. D. Anderson Cancer Center 1515 Holcombe Boulevard Houston Texas 77030; ^4^ Department of Biomathematics The University of Texas M. D. Anderson Cancer Center 1515 Holcombe Boulevard Houston Texas 77030

**Keywords:** margin, PTV, conformal stereotactic radiotherapy, intracranial

## Abstract

The planning target volume (PTV) includes the clinical target volume (CTV) to be irradiated and a margin to account for uncertainties in the treatment process. Uncertainties in miniature multileaf collimator (mMLC) leaf positioning, CT scanner spatial localization, CT‐MRI image fusion spatial localization, and Gill‐Thomas‐Cosman (GTC) relocatable head frame repositioning were quantified for the purpose of determining a minimum PTV margin that still delivers a satisfactory CTV dose. The measured uncertainties were then incorporated into a simple Monte Carlo calculation for evaluation of various margin and fraction combinations. Satisfactory CTV dosimetric criteria were selected to be a minimum CTV dose of 95% of the PTV dose and at least 95% of the CTV receiving 100% of the PTV dose. The measured uncertainties were assumed to be Gaussian distributions. Systematic errors were added linearly and random errors were added in quadrature assuming no correlation to arrive at the total combined error. The Monte Carlo simulation written for this work examined the distribution of cumulative dose volume histograms for a large patient population using various margin and fraction combinations to determine the smallest margin required to meet the established criteria. The program examined 5 and 30 fraction treatments, since those are the only fractionation schemes currently used at our institution. The fractionation schemes were evaluated using no margin, a margin of just the systematic component of the total uncertainty, and a margin of the systematic component plus one standard deviation of the total uncertainty. It was concluded that (i) a margin of the systematic error plus one standard deviation of the total uncertainty is the smallest PTV margin necessary to achieve the established CTV dose criteria, and (ii) it is necessary to determine the uncertainties introduced by the specific equipment and procedures used at each institution since the uncertainties may vary among locations.

PACS number(s): 87.53.Kn, 87.53.Ly

## I. INTRODUCTION

The goal of radiotherapy is to deliver a prescribed dose to the clinical target volume (CTV) while minimizing the dose to neighboring normal tissues. This is especially important in treating brain lesions because of the functional importance of brain tissues and structures, and the possibility of neurological complications when they are irradiated.^1^ Achieving this goal requires an understanding of the uncertainties inherent in the treatment planning and delivery process to generate a planning target volume (PTV) margin.^2^ ^,^ ^3^ By identifying and quantifying these uncertainties, it may be possible to determine the required margin to ensure adequate treatment of the CTV while minimizing toxicity to nearby normal tissues. This work examines the potential uncertainties for conformal stereotactic radiotherapy (SRT) procedures for intracranial lesions.

The Gill‐Thomas‐Cosman (GTC) noninvasive frame is utilized for SRT patient repositioning in order to minimize positional variations between fractions. Previous studies have shown that the repositioning uncertainty is approximately 0.4 mm.^4^ ^,^ ^5^ The miniature multileaf collimator (mMLC) was designed to improve small field dose conformation, especially in cases of irregularly shaped lesions.^6^ ^,^ ^7^ The manufacturer has given a leaf positioning accuracy of ≤0.5 mm, but this uncertainty should be verified prior to clinical use. Image fusion, which combines the CT and MRI images into a single image, offers the combination of improved target visualization of MRI with the superior spatial accuracy of CT.^8‐10^ However, previous studies have reported that the uncertainty in the CT‐MRI fusion process may be as large as 1.0 mm.^11^

The purpose of this work was to quantify uncertainties in mMLC leaf precision, GTC head frame reproducibility, and spatial localization of the image fusion software, and to use Monte Carlo calculations for various margin and fraction combinations for a large patient population from which we could recommend a minimum PTV margin for conformal SRT.

## II. MATERIALS AND METHODS

### A. Leaf positioning

The precision of mMLC leaf positioning was measured relative to the axis of collimator rotation. A sheet of XV film (Eastman Kodak, Rochester, NY) was placed at isocenter perpendicular to the beam with 1.5‐cm solid water for buildup. This setup was used to provide enough buildup to reach electronic equilibrium without significantly blurring the field edges. Lines were drawn on the film jacket corresponding to profiles under leaf pairs 2, 9, 16, 23, and 30 and marked for later use in film scanning and profile measurements.

The photon jaw opposing the leaf bank to be tested was set to +5.0 cm from the central axis (CAX), while the photon jaw on the same side as the leaves was set to –0.1 cm. The film was exposed for 20 monitor units (MU) because the XV film response is approximately linear in low‐dose regions. Therefore, 50% dose was approximately the same as 50% optical density (OD), which implied that a dose response curve for the film was not needed.

**Figure 1:**
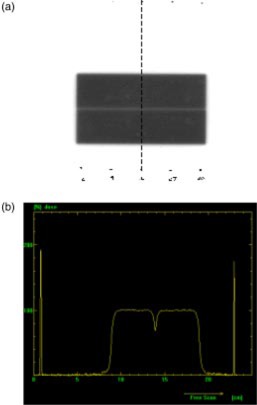
(Color) Jaw offset measurement for determining leaf position. (a) Film used in a jaw offset measurement. The profile alignment points are clearly visible. The dotted line denotes the profile shown in (b). (b) Profile from leaf pair 23 in the film. The leaf position being measured can be referenced to the photon jaw position to determine position relative to the collimator axis of rotation.

The collimator was then rotated 180° without moving either photon jaw or the film, and the field was exposed for another 20 MU. The films were scanned and each profile was normalized to its maximum dose. An example film image and measured profile are shown in Figs. 1(a) and 1(b). The average full width half maximum (FWHM) was determined as the distance between the 50% dose points. Half of the FWHM was used as the photon jaw offset from the collimator axis of rotation. The uncertainty in measurement of the profile width was 0.1 mm as determined previously by repeated measurements on a fixed field width. This process was performed four times to determine the average measured photon jaw offset.

The photon jaw positioned at –0.1 cm was then fully retracted while leaving the +5.0 cm photon jaw fixed. Another film jacket with profiles marked was placed at isocenter and the leaf bank opposite the set photon jaw was set to +5.0 cm. The film was exposed for 20 MU and the profile width for each leaf pair was measured. The photon jaw offset previously measured was subtracted from this profile width to give the leaf position relative to the axis of rotation. This procedure was repeated for both mMLC leaf banks at 1 cm increments across the full range of motion.

**Figure 2:**
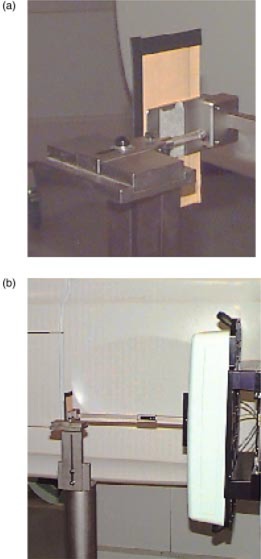
(Color) Measurement setup for gravitational effects on leaf positioning showing (a) the radiopaque target ball and film holder and (b) relation of the apparatus to the mMLC and gantry.

To evaluate the effect of gravity, leaf positioning measurements were made with the gantry at 90° and 270°. This placed the direction of leaf travel parallel to the gravitational field. A mechanical chanical isocenter stand (MIS) was used to verify laser alignment to isocenter prior to stereotactic radiosurgery and radiotherapy procedures. The MIS consists of a rigid metal pole that is secured to the floor and is capable of holding an alignment insert. The MIS was attached to the floor, and a stereotactic radiopaque target ball was placed at isocenter. The stereotactic quality assurance (QA) film holder was attached to the bottom of the mMLC. The setup for these measurements is shown in Figs. 2(a) and 2(b). The holder is designed to hold a small piece of film, approximately 5 cm×15 cm, below the target ball for QA measurements. When the film and target ball are exposed to radiation, the location of the target ball within the mMLC field can be determined.

**Figure 3:**
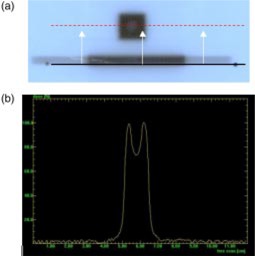
(Color) (a) Film used to measure gravitational effects on leaf positioning. The open leaf pair at the bottom is used to align the scanner profile. The profile is then shifted to the center leaf pair to pass under the target ball. (b) The dip in the center is caused by the target ball. Gravitational effects on leaf positioning can be determined by locating the target ball with respect to the field edges at gantry positions of 0°, 90°, and 270°.

With the gantry at 0°, the mMLC was set to a $1.2\times 1.2{\text{cm}}^{2}$ field. One leaf pair outside of the square field was opened to use as an alignment reference. To help align the film with the mMLC leaf, a line was drawn on the jacket of a piece of film. This line was placed parallel to the edge of the open leaf pair and then translated so that it passed through the center of the target ball. This ensured that the profile passed under the center leaf of each bank. By evaluating the location of the target ball with respect to the field, the repositioning of the leaves with the gantry at 90° could be measured. An example film image and profile through the target ball are shown in Fig. 3.

A piece of film was placed in the holder and exposed for 20 MU. The gantry was then rotated to 90° without moving the mMLC leaves. A new piece of film was placed in the film holder and again exposed for 20 MU. Changes in the position of the target ball with respect to the field from this profile compared to the position of the target ball in the first profile would be dependent only on the gantry sag. The mMLC was fully opened, the field reformed, and another film exposed. The position of the target ball with respect to the third field was now dependent solely on leaf positioning when compared to the second film. The gantry was then returned to 0° and the mMLC field retracted and reset. Another film was exposed. This process was repeated for a gantry angle of 270°. The difference between films #2 and #3 represented leaf position error due to gravity. This was also true for films #1 and #4.

### B. Image fusion software

**Figure 4:**
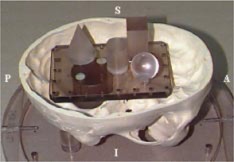
(Color) QA phantom used in the image fusion evaluation. The internal geometric structures are clearly visible.

The Radionics Image Fusion Software (version 2.0) was used to determine the uncertainty in the target coordinates determined by the image fusion process. During this evaluation, spatial uncertainty in the CT scan was also determined. We used a skull phantom supplied by Radionics for the purpose of evaluating the spatial localization accuracy of the imaging systems. The phantom, shown in Fig. 4 with the top removed, contains four geometric structures (cone, cylinder, sphere, and cube) located at fixed positions within the phantom. Radionics supplies the stereotactic coordinates for the top center of each of the objects to compare with the calculated coordinates from the imaging system.

Ten CT scans of the phantom were acquired on n PQ5000 CT scanner (Marconi Medical Systems, Cleveland, Ohio) with 1.5‐mm slice spacing and thickness, 32‐cm FOV, and 512×512 matrix, resulting in a pixel size of 0.63 mm×0.63 mm. The scan range was chosen to entirely cover the geometric structures within the phantom. A MRI scan of the phantom was made and fused with the multiple CT scans. Initial fusion alignment was performed using user‐selected points on each of the scan data sets. The final image fusion was performed using an intensity‐matching algorithm. The MRI scan was performed with a T1 weighted spin echo sequence, 1.5‐mm slice spacing and thickness, 25‐cm FOV, and a 256×256 matrix, resulting in a pixel size of 0.98 mm×0.98 mm.

We began the image fusion process by selecting four corresponding landmark points on one CT image set and one MRI image set. Once the points were selected, the software aligned the landmarks to use as a starting point in the final fusion. Once the landmark alignment was selected, the images were fused. The initial selection of points is only used as a starting point in the fusion process and the transformation required to align the selected points is provided to the user before the final fusion is performed. Thus, the transform required to align the user selected points can be compared to the final fusion transform provided by the software. It should be noted, based on evaluation at our institution, that with the intensity‐matching algorithm, the initial selection of points has little effect on the final transform used to fuse the image sets. Although this evaluation is performed in the idealized situation of a phantom, it at least gives an initial quantitative estimate of the uncertainty introduced by the fusion process.

Six points were chosen for evaluation–tip of the cone, top center of the cube, left‐anterior‐superior corner of the cube, top center of the sphere, center of the sphere, and top center of the cylinder. Coordinates for points not provided by Radionics were determined by opening the phantom and measuring the dimensions of each structure. The unknown coordinates could then be determined relative to the known coordinates. With the fusion complete, the fused image set was evaluated in XPlan, a commercial treatment planning system for geometric conformal stereotactic radiosurgery and radiotherapy. Each of the points chosen for evaluation was identified, and the coordinates of each point were recorded. The point selection was evaluated by three different qualified users to determine if there was a user dependence. There was no measurable difference in the point coordinates obtained by the three different users. This process was repeated until the MR image set had been fused with each CT image set. The mean and standard deviation of the distribution were determined from the measured coordinates in each direction (AP, LAT, and VERT) for all six points.

The coordinates for each of the points were then determined on the 10 CT image sets alone. The mean and standard deviation of the distribution were determined from the measured coordinates in each direction for all six points. The CT coordinates were compared to the given stereotactic coordinates, uncertain how to determine the CT scan. The mean coordinate and standard deviation of each corresponding point‐direction combination from the two measured distributions (fused and CT alone) were then compared. The difference in the means was determined, and the standard deviations were added in quadrature. The measured difference was the result of a combination of the spatial inaccuracy of the MR scanner and the image fusion process. Since the local spatial inaccuracy for a particular MR scanner is not known, an evaluation must be performed for each MR scanner used in treatment planning. In other words, the margin applied to the CTV will depend on the scanner used in the imaging process.

### C. GTC frame reproducibility

**Figure 5:**
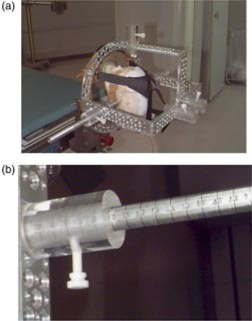
(Color) (a) MDACC alignment frame and (b) one of the depth measurement rods. The frame uses five tattoo points to align patient anatomy for treatment. The depth rods are marked with a millimeter resolution scale for depth measurements to evaluate patient positioning reproducibility.

Reproducibility of the GTC frame was determined using a method described by Kooy *et al*.^5^ The measured uncertainty in GTC repositioning is a combination of two quantities: (1) the actual repositioning uncertainty and (2) the uncertainty in the measurement itself. Our institution's in‐house alignment frame and depth‐measurement rod are shown in Fig. 5. The frame is attached to the GTC frame and is used to mark five alignment points on the patient's skin using a metal rod with a felt‐pen marker at the end. There are two superior points, one anterior point, and two lateral points. Once it has been determined that there are no problems with the placement of the frame with respect to the target, permanent tattoos are placed at each point and the alignment frame is removed. The patient then undergoes CT imaging. These tattoos are used for repositioning before each treatment fraction. For each subsequent setup, pointed rods are used to align the tattoos with the alignment frame. Alignment of the points with the frame corrects for both translation and rotation of the patient anatomy. The alignment rods fit snugly into the guide holes assuring highly accurate and reproducible positioning. Each rod is marked with a millimeter resolution scale to measure the depth to the patient surface. This allows for the reproducibility to be quantified compared to the standard clinical practice of just aligning the points.

Similar to the Radionics depth confirmation helmet, commonly used in SRT patient repositioning, the MDACC alignment frame has two laterally opposed measurement points. A lateral shift of the cranium would cause the lateral measurements to change coherently. Because the cranial width remains constant, the sum of the lateral measurements should remain constant. Therefore, the uncertainty in the sum of the left and right measurements $\left(\sigma_{L+R}\right)$ depends only on the measurement uncertainty $\left(\sigma_{M}\right)$ and not the positioning uncertainty of the patient. This can be assumed since the uncertainty in the sum of the left and right measurements $\left(\sigma_{L+R}\right)$ is the standard deviation of the distribution of the sum of the left and right lateral measurements. The measurement uncertainty is the uncertainty in the sum of the lateral measurements divided by the square root of two (i.e., $\sigma_{M}=\sigma_{L+R}/\sqrt{2})$.^5^ It is assumed that the measured uncertainty of each position is the quadrature sum of the positioning uncertainty and the measurement uncertainty. The measurement uncertainty can then be separated from the total measured uncertainty in each point to find the positioning uncertainty. The positioning uncertainty is given by

(1) $$\sigma_{p}=\sqrt{\sigma_{\text{tot}}^{2}-\sigma_{M}^{2}},$$

where $\sigma_{p}$ is the positioning uncertainty for a particular point, $\sigma_{\text{tot}}$ is the measured total uncertainty for that point, and $\sigma_{M}$ is the measurement uncertainty.

The evaluation of the GTC frame was performed on a patient undergoing treatment. Measurements for each of the points were made during setup for each of 14 fractions.

### D. Monte Carlo calculation for target coverage

Evaluating the effects of margin selection on CTV dose for a large patient population would require randomly shifting the CTV based on the measured uncertainties in each direction and then evaluating the CTV dose/volume relation. It would be impractical to perform measurements on a large number of “patients” using standard dosimetry techniques since a 3D dose distribution would be required for each CTV shift. Therefore, the Monte Carlo program was written to evaluate the margin and fraction combinations for a large number of patients.

The simulation makes a number of basic assumptions that differ to some extent from physical reality. However, the simplified target geometry and dose distribution will still provide useful information on the effects of margin and fraction combinations. We assumed that any point within the PTV received 100% of the prescribed dose, and that the dose outside of the PTV fell off linearly to zero dose. Outside of this falloff region the dose was assumed to be zero. We also performed the calculations based on a 4‐cm diameter spherical CTV All of the measured uncertainties determined for each of the components considered in this work were combined to generate an overall uncertainty in each of the three independent directions. The random uncertainties were assumed to follow Gaussian probability distributions and were therefore added in quadrature, assuming no correlation, to arrive at the final uncertainty to be applied to the PTV margin. The systematic errors were added linearly in each direction.

The number of fractions to be simulated is selected at the start of the CTV coverage evaluation. A number of points are then randomly selected within the CTV to approximate the CTV A variable margin is applied to the CTV in each direction to generate a PTV. A random shift is generated, based on measured uncertainties, in each direction and applied to each CTV point. The dose to each point is then calculated. Points located within the PTV are assigned a dose value of 1 for that particular fraction. Points located outside of the dose falloff region are assigned a dose value of 0 for that particular fraction. Points located in the dose falloff region are assigned a dose value dependent on their distances from the surface of the PTV. A dose gradient of $20\%{\text{mm}}^{-1}$ exterior to the PTV was used since it is approximately the same dose gradient as the mMLC penumbra.

The region enclosed by the PTV, and therefore the volume of 100% dose, is approximated as an ellipsoidal volume defined by the equation

(2) $$\frac{x^{2}}{a^{2}}+\frac{y^{2}}{b^{2}}+\frac{z^{2}}{c^{2}}⩽1,$$

where *x, y,* and *z* are the coordinates of the CTV point after the random shifts are applied and *a, b,* and *c* are the CTV radius plus the margin along the *x, y,* and *z* axes, respectively. Points with coordinates satisfying this equation are assigned a dose value of 1 for that particular treatment fraction.

The region of zero dose is defined as the space of points whose coordinates satisfy the equation

(3) $$\frac{x^{2}}{{\left(a+1/g\right)}^{2}}+\frac{y^{2}}{{\left(b+1/g\right)}^{2}}+\frac{z^{2}}{{\left(c+1/g\right)}^{2}}⩾1,$$

where *g* is the dose gradient. Thus, 1/*g* is the distance between the region of 100% dose and the region of zero dose. Points with coordinates satisfying this equation are assigned a dose value of zero.

If the point coordinates do not satisfy either of the above conditions, then the point lies in the region of the linear dose falloff. The point coordinates approximately satisfy an equation of the form

(4) $$\frac{x^{2}}{{\left(a+d\right)}^{2}}+\frac{y^{2}}{{\left(b+d\right)}^{2}}+\frac{z^{2}}{{\left(c+d\right)}^{2}}=1,$$

where *d* is the distance from the surface of the volume of 100% dose to the surface satisfied by the point coordinates. The dose at this point can be calculated as

(5) dose=1.0−d*g.

A bisection method was used to determine the value of *d* that was used in Eq. (4).

The CTV shift was resampled for each fraction in the “treatment” and the dose to each point for that fraction was calculated. The dose for each point was summed over the total number of fractions and then divided by the number of fractions in the treatment. This resulted in the average dose for each point being a percentage of the intended PTV dose. The dose information was then used to generate a cumulative dose volume histogram (cDVH) for the CTV. This represented a treatment simulation of a single patient. The entire process (excluding the random sampling of target points) was repeated 1000 times, representing the treatment of 1000 patients.

The cDVH's were used to determine a mean cDVH and confidence intervals for the mean cDVH. The cDVH's were also used to create histograms of the dose/coverage relationship. One histogram plots the distribution of frequencies of deviations from 100% target coverage (i.e., CTV dose where target coverage drops below 100%). The other histogram plots the distribution of frequencies of target volume covered by 100% of the PTV dose. The mean cDVH, confidence intervals, and histograms show CTV coverage in a population of patient treatments over a number of combinations of PTV margin and number of treatment fractions.

## III. RESULTS AND DISCUSSION

### A. Leaf positioning

**Figure 6:**
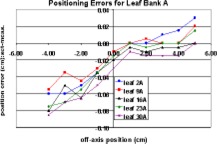
(Color) Measured “A bank” leaf positioning errors.

**Figure 7:**
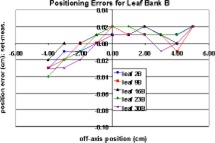
(Color) Measured “B bank” leaf positioning errors.

Figures 6 and 7 show the measured positioning errors versus set position for leaves on the *A* and *B* banks, respectively. Positive set position numbers indicate leaf positions on the same side of the central axis (CAX) as the leaf bank being examined. The positioning error for the *A* bank of leaves exceeded the manufacturer's stated positioning accuracy for positions greater than 1 cm across the central axis. The *B* bank of leaves met the manufacturer's specifications at all positions within the range of motion.

The full range of mMLC leaf motion was divided into three regions: (1) fully extended to –2.00 cm, (2) –2.00 cm to +2.00 cm, and (3) +2.00 cm to fully retracted. A leaf calibration factor can be applied independently to each of these regions. Therefore, adjustments can be made in the regions where the positioning error exceeds the manufacturer's specifications without affecting regions where the positioning error meets the specifications. Since these modifications must be made by the manufacturer, and the manufacturer will only guarantee a positioning uncertainty of ±0.5 mm, it can be assumed that the post‐modification positioning error will be ≤0.5 mm. Assuming worst case scenario, the leaf positioning uncertainty was incorporated in the Monte Carlo calculations as a systematic error of 0.5 mm and an additional random error uniformly distributed between 0.0 and 0.14 mm. The random error accounts for the positioning uncertainty at nonzero gantry angles and the uncertainty in the measurements themselves.

### B. Image fusion software

The measured coordinates of the selected points within the QA phantom were compared to the known coordinates for each of the points. Table I shows the measured uncertainties [mean ±1 standard deviation (SD)] between the fused image sets and the CT image sets for each point in each direction along with the average measured uncertainty in each direction. The average value in each direction was determined by summing the values in that direction for each point and dividing by the number of points. The standard deviations were added in quadrature assuming no correlation. The average values were used in the Monte Carlo calculations.

**Table I:** Measured uncertainties for image fusion.

Structure	AP	LAT	VERT
Top of cube	–0.4±0.2 mm	0.0±0.2 tmm	–0.3±0.7 mm
Corner of cube	0.0±0.3 mm	–0.4±0.2 mm	–0.2±0.5 mm
Cylinder	–0.5±0.2 mm	0.0±0.1 mm	–0.1±0.8 mm
Cone	–0.5±0.2 mm	–0.2±0.2 mm	0.9±0.9 mm
Top of sphere	0.0±0.3 mm	0.2±0.2 mm	0.8±0.7 mm
Center of sphere	–0.2±0.2 mm	0.1±0.2 mm	–0.4±0.7 mm
Average	–0.2±0.1 mm	0.0±0.1 mm	0.1±0.3 mm

### C. GTC frame reproducibility

Table II shows the measurements for a patient under treatment over four weeks. The resulting measurement error was 0.74 mm. The results show positioning errors (after removing the measurement error) in only the lateral dimensions with magnitudes of 0.3 mm and 0.4 mm on the left and right sides, respectively. These results agree well with the results of Kooy *et al*.^5^ who determined a measurement error of 0.71 mm and a lateral positioning uncertainty of 0.35 mm.

**Table II:** Measured GTC respositioning uncertainties

#	Anterior	Left	Right	Left vert	Right vert	Left+right
1	9.5 cm	12.7 cm	14.1 cm	14.5 cm	14.8 cm	26.8 cm
2	9.5 cm	12.9 cm	14.1 cm	14.5 cm	14.8 cm	27.0 cm
3	9.5 cm	12.9 cm	14.0 cm	14.5 cm	14.8 cm	26.9 cm
4	9.6 cm	12.8 cm	14.0 cm	14.4 cm	14.8 cm	26.8 cm
5	9.6 cm	12.9 cm	14.2 cm	14.5 cm	14.7 cm	27.1 cm
6	9.6 cm	12.8 cm	14.2 cm	14.5 cm	14.8 cm	27.0 cm
7	9.5 cm	12.9 cm	14.1 cm	14.5 cm	14.7 cm	27.0 cm
8	9.5 cm	12.9 cm	14.2 cm	14.5 cm	14.8 cm	27.1 cm
9	9.5 cm	12.9 cm	14.0 cm	14.5 cm	14.7 cm	26.9 cm
10	9.5 cm	12.9 cm	14.0 cm	14.6 cm	14.8 cm	26.9 cm
11	9.6 cm	13.0 cm	14.0 cm	14.4 cm	14.8 cm	27.0 cm
12	9.6 cm	12.9 cm	14.0 cm	14.5 cm	14.7 cm	26.9 cm
13	9.6 cm	13.0 cm	14.1 cm	14.5 cm	14.7 cm	27.1 cm
Standard deviation	0.052 cm	0.080 cm	0.083 cm	0.049 cm	0.051 cm	0.104 cm $\left(\sigma_{L+R}\right)$
Measurement error	0.074 cm $\left(\sigma_{M}\right)$
Position error	0.00 cm	0.03 cm	0.04 cm	0.00 cm	0.00 cm

The recommendations of this work are based on measurements made on a single patient. However, the results agree well with measurements made by Kooy *et al*.^5^ on a larger sample of subjects. As a conservative measure, the positioning error measured for the lateral dimension will be applied to all three dimensions as a random error in the determination of the PTV margin.

### D. Monte Carlo calculation

Table III shows the quantified uncertainties for each component and the total uncertainty for each MRI unit. The total uncertainty was used in the Monte Carlo calculations.

**Table III:** Summary of measured individual and combined uncertainties.

	AP	LAT	VERT
Component systematic	±1 SD	systematic±1 SD	systematic±1 SD
Leaf positioning	0.5±0.14 mm	0.5±0.14 mm	0.5±0.14 mm
GTC repositioning	0.0±0.35 mm	0.0±0.35 mm	0.0±0.35 mm
CT	0.3±0.1 mm	0.3±0.0 mm	0.1±0.2 mm
Image fusion	0.2±0.1 mm	0.0±0.1 mm	0.0±0.3 mm
Total uncertainty	1.0±0.4 mm	0.8±0.4 mm	0.6±0.5 mm

**Table IV:** Summary of Monte Carlo results for five fractions

Margin	Min. dose range	99th %tile dose	Target vol. range	99th %tile vol.
None	69%–91%	71%	86%–94%	88%
Systematic	87%–99%	88%	94%–100%	95%
Systematic+1 SD	94%–100%	95%	96%–100%	97%

**Table V:** Summary of Monte Carlo results for 30 fractions.

Margin	Min. dose range	99th %tile dose	Target vol. range	99th %tile vol.
None	77%–86%	78%	84%–91%	85%
Systematic	93%–98%	93%	93%–98%	94%
Systematic+1 SD	97%–100%	98%	96%–100%	97%

**Figure 8:**
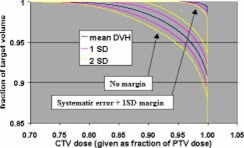
(Color) Mean DVH (blue), ±1σ interval (pink), and ±2σ interval (yellow) for five fractions with no margin and the systematic error plus one standard deviation margin.

**Figure 9:**
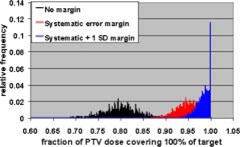
(Color) Minimum target dose histograms for five fraction treatment with no margin (black), systematic error margin (red), and systematic error plus one standard deviation margin (blue).

**Figure 10:**
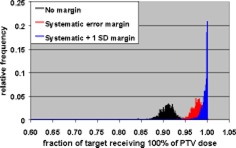
(Color) Histograms of target volume receiving 100% of PTV dose for five fraction treatment with no margin (black), systematic error margin (red), and systematic error plus one standard deviation margin (blue).

**Figure 11:**
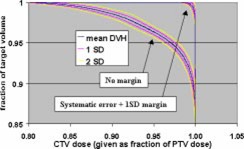
(Color) Mean DVH (blue), ±1σ interval (pink), and ±2σ interval (yellow) for 30 fractions with no margin and the systematic error plus one standard deviation margin.

**Figure 12:**
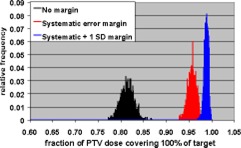
(Color) Minimum target dose histograms for 30 fraction treatment with no margin (black), systematic error margin (red), and systematic error plus one standard deviation margin (blue).

**Figure 13:**
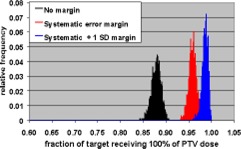
(Color) Histograms of target volume receiving 100% of PTV dose for 30 fraction treatment with no margin (black), systematic error margin (red), and systematic error plus one standard deviation margin (blue).

The Monte Carlo calculations were used to evaluate CTV coverage for a number of PTV margins and treatment fractionation schemes. Calculations were made for 5 and 30 fraction treatments, and each fractionation scheme was evaluated using no margin, just the systematic error margin, and the systematic margin plus one standard deviation random uncertainty. The tests were performed with no margin to demonstrate the reduction in CTV coverage if the measured uncertainties are not taken into account. These two fractionation schemes were chosen since these are the only two currently used in SRT treatments at our institution. Single‐fraction treatments were not addressed since the GTC frame is not used for those treatments. The results of the Monte Carlo simulations are shown in Figs. 8–13.

A satisfactory dose/volume relation is considered a minimum CTV dose of greater than or equal to 95% of the PTV dose while delivering 100% of the PTV dose to greater than or equal to 95% of the CTV. Even if a 5% dose inhomogeneity (i.e., a minimum dose that is at least 95% of the prescribed dose) is specified in the inverse planning software, the calculated inhomogeneity is generally greater than that due to constraints on normal tissue doses and limitations of the planning system. The 5% inhomogeneity criterion allows for the planning system to generate a plan with an inhomogeneity greater than 5% that is still acceptable.

Tables IV and V show summaries of the Monte Carlo results for 5 and 30 fractions, respectively. Examination of the 99th percentile values for minimum CTV dose and fraction of CTV receiving the PTV dose shows that a margin of the systematic uncertainty plus one standard deviation in each direction results in a satisfactory CTV dose/volume relation for both 5 and 30 fraction treatments. This means that there is a 99% confidence that the minimum CTV dose is ≥95% of the PTV dose and that ≥95% of the CTV receives the full PTV dose.

## IV. CONCLUSIONS

The purpose of this work states that it is possible to quantify uncertainties in the image fusion software, mMLC leaf positioning, and GTC frame repositioning and combine these uncertainties to determine a PTV margin so that the entire CTV receives clinically acceptable coverage. However, it is necessary for each institution to quantify these uncertainties.

The CTV dose was evaluated for a number of margin and fractionation scheme combinations using a simple Monte Carlo simulation. Based on the Monte Carlo calculations, a margin of the systematic uncertainty plus one standard deviation in each direction is recommended for treatments of both 5 fractions and 30 fractions. This margin would be sufficient to achieve a minimum CTV dose greater than 95% of the PTV dose, while delivering 100% of the PTV dose to greater than 95% of the CTV

This work confirms that it is possible to quantify uncertainties in the treatment planning and delivery process and combine these uncertainties to generate a PTV margin such that the CTV receives the prescribed dose. However, the application of these results is dependent on the physician evaluating the results. Satisfactory dose/volume relations will depend on the physician to determine the necessary margin and resulting CTV dose for a particular fractionation scheme.
